# Supplementary material for: Transmission of heat modes across a potential barrier
Source: Nat Commun. 2017 Dec 21;8:2251. doi: 10.1038/s41467-017-02433-z (PMC5740138; doi:10.1038/s41467-017-02433-z)
Supplement: Supplementary file 1 — Supplementary Information [file 41467_2017_2433_MOESM1_ESM.pdf]

Supplementary Note 1: Eliminating bulk thermal transport

Here we present a second sample studied in order to probe if any contribution to the heat measured by the quantum dot was propagating through the bulk and not only through the edge modes. The device is very similar to the one from the main text. A deflector gate is placed before the quantum dot that allows to redirect the neutral modes to the ground directly. The results of the thermoelectric current measured versus the plunger gate voltage for the two configurations, deflector open and close, are presented on Supplementary Figure 1. When the QPC is closed a net thermoelectric current is measured similarly to the main paper sample. Nevertheless, one can notice that the thermoelectric current is only positive, which is due to the fact that we used a spectrum analyzer, only sensitive to the absolute value of the signal. Conversely, when the deflector is open, no thermoelectric current is measured by the quantum dot, which shows that no measurable contribution arise from the bulk.

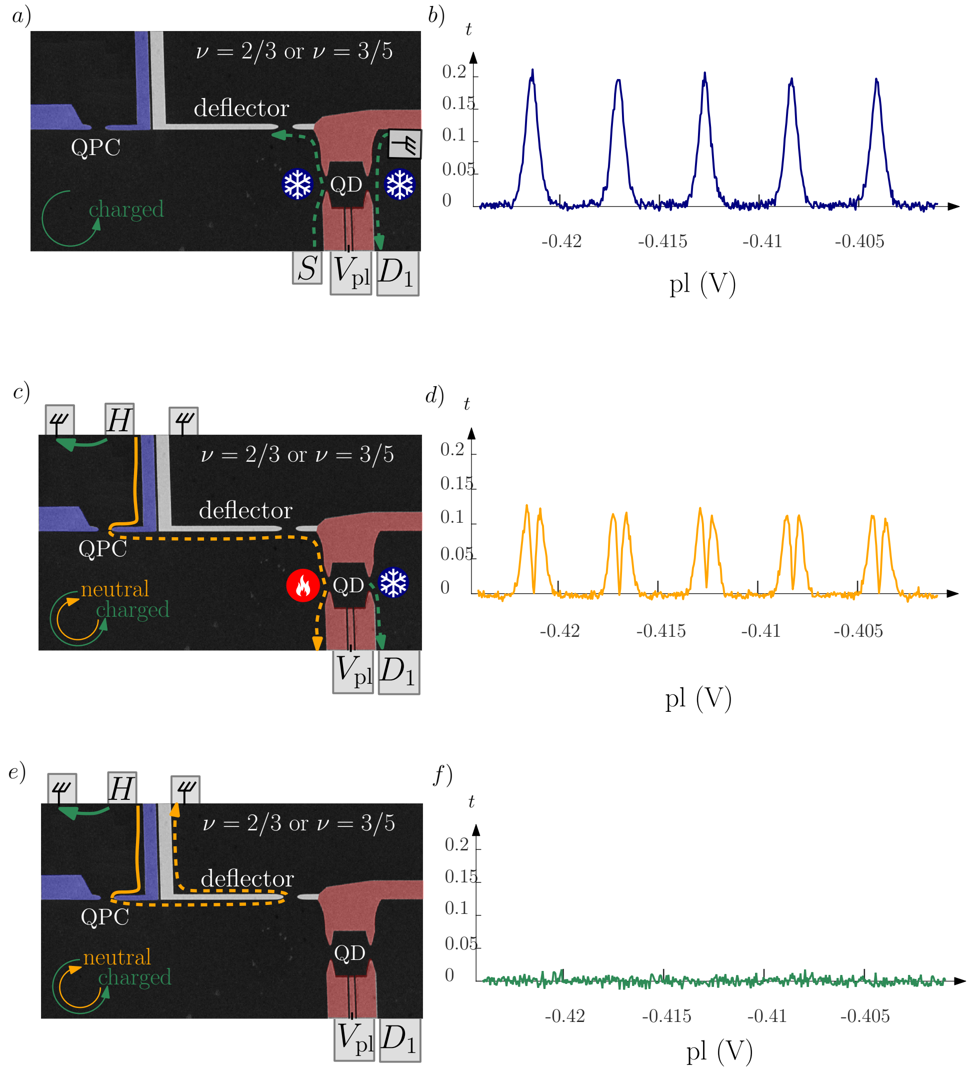

Supplementary Figure 1. Measurement of the second sample: a) Configuration corresponding to the measurement of the Coulomb blockade peaks. both sides of the quantum dot are at base temperature. Sourcing current from S results in transmission peaks measured in D1 when changing the plunger gate voltage, as visible on **b)**. **c)** Configuration corresponding to the measurement of the neutral transmission using the thermoelectric current created through the quantum dot when the deflector gate is closed. **d)** Evolution of the thermoelectric current across the quantum dot as function of the plunger gate voltage. **e)** Configuration corresponding to the measurement of the neutral transmission using the thermoelectric current created through the quantum dot when the deflector gate is open **f)** No thermoelectric current is measurable through the QD when the deflector is open showing that no heat transport is happening in the bulk of the sample.

Supplementary Note 2: Upstream neutral modes at other filling factors

In Supplementary Figures 2 we present thermoelectric measurements at other filling factors, $\nu = 2,\ 1,\ 2/3,\ 3/5,\ 2/5,\ 1/3$ together with the coulomb blockade (CB) conductance as function of the plunger gate. At all of the measurements we sourced AC voltage of $V_\mathrm{H} \sim 70\,\mu\mathrm{V_{RMS}}$ and scanned the plunger over a range of CB peaks where the QPC if fully open and the deflector gate is energized. At fillings $\nu = 2$, 2/5 and 1/3 there were no measurable thermoelectric voltage, consisting with the lack of upstream neutral modes at these states. At fillings $\nu = 1$, 2/3 and 3/5 we measure significant thermoelectric voltage consisting with the upstream neutral expected in the hole-conjugate states ($\nu = 2/3$ and 3/5). At filling $\nu = 1$ upstream heat mode was measured before [1, 2] and it was attribute to a state of $\nu = 2/3$ underlying the $\nu = 1$.

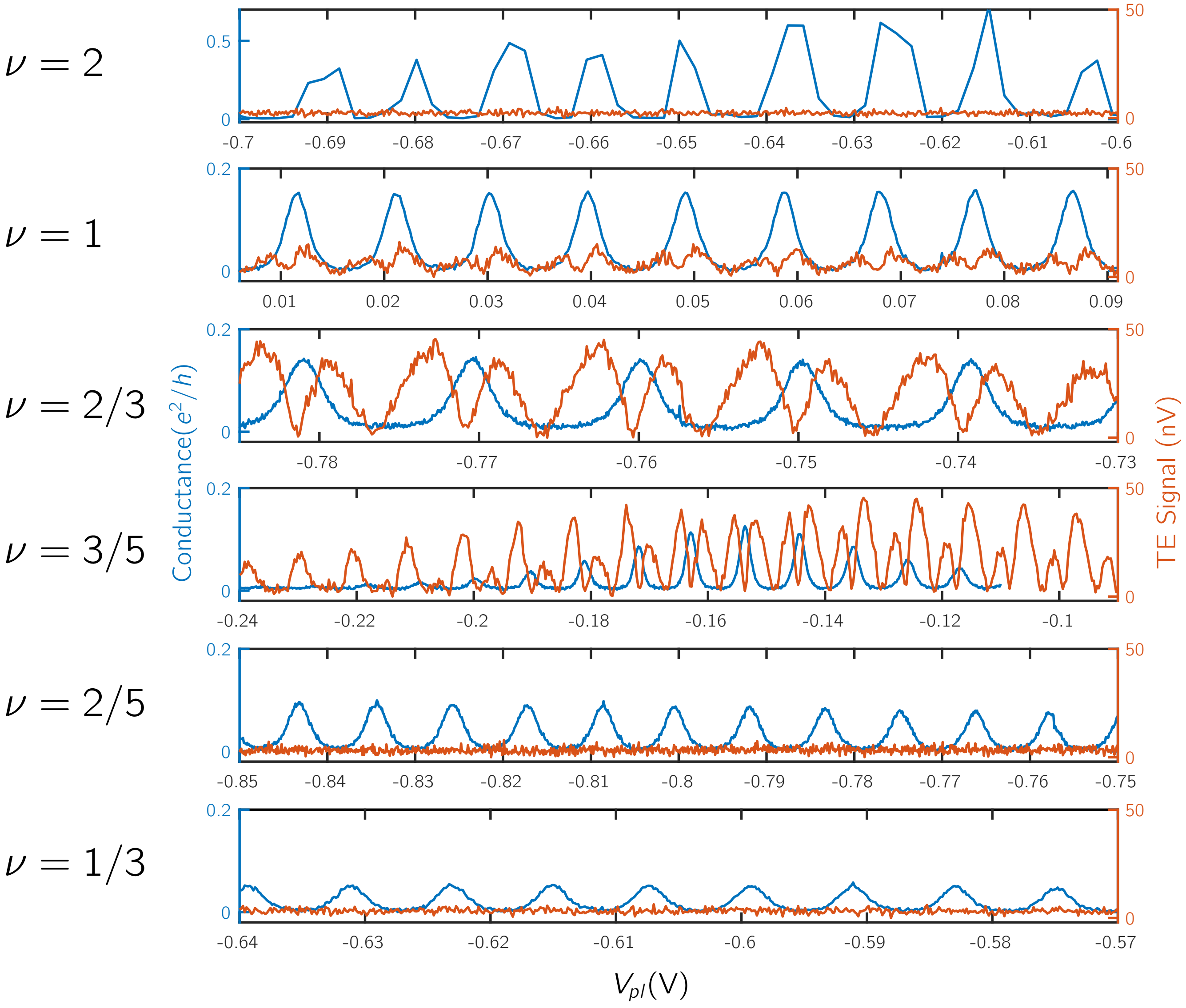

Supplementary Figure 2. Evolution of the Coulomb peaks and thermoelectric currents for several filling factors: Thermoelectric voltage at other filling factors, $\nu = 2,\ 1,\ 2/3,\ 3/5,\ 2/5,\ 1/3$. Blue curves - left axes - conductance through the QD tuned to Coulomb blockade regime. Orange curves - right axes - thermoelectric voltage at the same regime. Measured with the deflector gate energized and $V_\mathrm{H} \sim 70\,\mu\mathrm{V_{RMS}}$

Supplementary Note 3: Quantum Dot Coulomb diamonds

Here is presented the evolution of the conductance in the quantum dot as function of the voltage applied in S1 and the plunger gate. The extracted charging energy is $\sim 25\,\mu$eV which corresponds to an equivalent temperature of $\sim$ 290 mK.

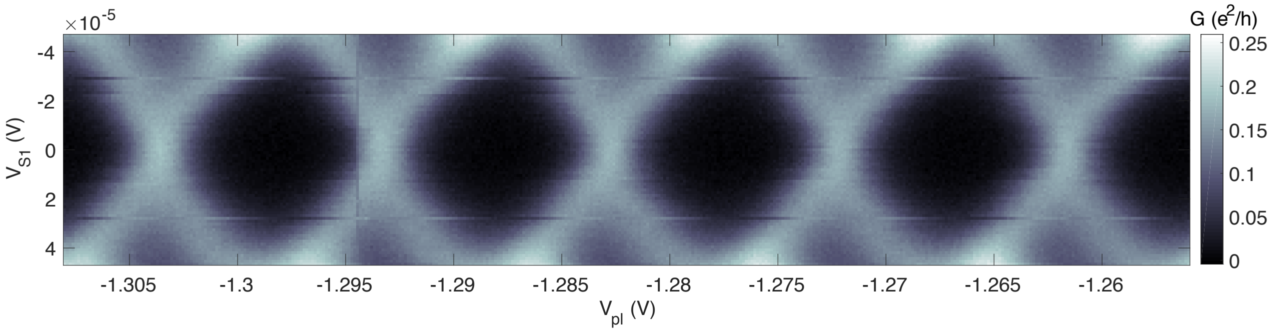

Supplementary Figure 3. Coulomb Diamonds: Evolution of the conductance of the quantum dot as function of V_{S1} and V_{pl}. Coulomb diamonds are visible with a typical charging energy of $25\,\mu$eV with corresponds to an equivalent temperature of $\sim$ 290 mK

Supplementary Note 4:Theoretical model

Fractional quantum Hall states at simple Landau level filling fractions like $\nu = 1/m$ with odd m could be described by the Laughlin wave function [3]:

$$\langle z_1, ..., z_n|\Psi_{\mathrm{L}}\rangle \propto \prod_{i<j}(z_i - z_j)^m \exp[-\frac{1}{4l_{\mathrm{B}}^2}\sum_i |z_i|^2], \tag{1}$$

where $z_i = x_i + iy_i$ are complex coordinates of electrons in the plane of two-dimensional electron gas and $l_{\mathrm{B}} = \sqrt{\hbar c/eB}$ is the magnetic length. This state features fractionally charged anyons that are created by the operator $\psi^\dagger(\zeta)$ defined via

$$\langle z_1, ..., z_n|\psi(\zeta)|\Psi_{\mathrm{L}}\rangle = \prod_i(\zeta - z_i)\langle z_1, ..., z_n|\Psi_{\mathrm{L}}\rangle. \tag{2}$$

The Laughlin wave function (1) has a great success in describing various aspects of the corresponding fractional quantum Hall states. It has also been shown to be very close to exact eigenstate by numeric calculations and shown to be a universal exact wave function for short range interactions.

For more complex filling fractions like $\nu = 2/3, 3/5$, etc., it is still not completely clear what is the structure the wave function. Here we focus on the $\nu = 2/3$ state and discuss consequences for other filling factors later. There are two points of view on the $\nu = 2/3$ state. One considers this state as a charge conjugate to the $\nu = 1/3$ state. In other words, holes in the completely filled Landau level $\nu = 1$ form a Laughlin condensate with $\nu = 1/3$. Another one considers $\nu = 2/3$ state as a $\nu = 1$ type condensate of fractional $e^* = 1/3$ quasi-particles on top of the $\nu = 1/3$ state. It can be described by the wave function:

$$|\Psi_{\frac{2}{3}}\rangle \sim \int J(\zeta, \bar{\zeta})d^M\zeta d^M\bar{\zeta}\prod_{i<j}(\zeta_i - \zeta_j)\exp[-\frac{1}{4ml_{\mathrm{B}}^2}\sum_i|\zeta_i|^2]\prod_i\psi^\dagger(\zeta_i)|\Psi_{\mathrm{L}}\rangle. \tag{3}$$

Note that the magnetic length of inner condensate is renormalized by $\sqrt{m}$. Such construction in general could describe various filling factors in the hierarchic manner [4].

We argue that the present experiment indicates that the second point of view is more appropriate. In order to investigate the edge structure of such state (3), we take into account that the energy scales are much lower than the fractional gap and use the effective low-energy theory [5, 6, 7] to describe the edge states. It has been shown [8] that the generic action for Abelian effective model of edge sates can be always cast in the form:

$$S[\phi_s] = \frac{1}{4\pi}\sum_s\int dtdx[\sigma_s D_t\phi_s D_x\phi_s - v_s(D_x\phi_s)^2 + Q_s\epsilon^{\mu\nu}a_\mu\partial_\nu\phi_s], \tag{4}$$

where $\mu = x, t$, $\sigma_s = \pm 1$ denotes chirality of the corresponding eigenmode, and covariant derivative $D_\mu \phi_s = \partial_\mu \phi_s + \sigma_s Q_s a_\mu$ depends on the couplings Q_s of the corresponding modes to the external electro-magnetic potential a_μ. The charge density operator in such theory is expressed in terms of boson fields as $\rho_s = (\sigma_s Q_s/2\pi)\partial_x \phi_s$. There has been proposed an edge reconstruction picture [14] as shown in Fig S3. Here we follow this idea and propose a particular model with $\hat{Q} = (1/\sqrt{3}, 1/\sqrt{3}, 0, 0)^T$, $\sum_s \sigma_s Q_s^2 = \nu$ and electron fields:

$$\phi_1 = \sqrt{3}\phi_c + \sqrt{\frac{3}{2}}\phi'_n + \frac{1}{\sqrt{2}}\phi''_n \tag{5a}$$

$$\phi_2 = \sqrt{3}\phi_c + \sqrt{2}\phi''_n \tag{5b}$$

$$\phi_3 = \sqrt{3}\phi_c - \sqrt{\frac{3}{2}}\phi'_n + \frac{1}{\sqrt{2}}\phi''_n \tag{5c}$$

Note that such model differs from proposed in Ref. [14] only in the degenerate subspace of the neutral modes. In the limit of strong interactions the velocity of charged mode is large $v_c \gg v'_n, v''_n$. We speculate that the width of inner reconstructed modes is also large $\xi/\xi_0 \gg 1$. This is consistent with the fact that the correlation length in the inner condensate of (3) is larger. Statistical phases for electronic excitation are indeed fermionic $\theta_{\alpha\beta} = \pi K_{\alpha\beta}$, $\alpha, \beta = 0, ..., 3$.

$$\hat{K} = \begin{pmatrix} 3 & 0 & 0 & 0 \\ 0 & 1 & 2 & 4 \\ 0 & 2 & 1 & 2 \\ 0 & 4 & 2 & 1 \end{pmatrix} \tag{6}$$

so that electron vectors form an integral lattice that has a hexagonal projection on the neutral sector. Quasi-particles must have single-valued wave functions as, e.g., Laughlin quasi-particles (2) discussed above. In the effective model language this translates into integer statistical phases with respect to all electronic excitation. In other words, quasi-particles form a dual lattice:

$$\chi_n = \frac{n_0}{\sqrt{3}}\phi_0 + \frac{n_1 - n_2 + n_3}{\sqrt{3}}\phi_c + \frac{n_3 - n_1}{\sqrt{6}}\phi'_n + \frac{n_1 - 2n_2 + n_3}{\sqrt{2}}\phi''_n \tag{7}$$

The charges of the corresponding excitation are $e^*_n = (n_0 + n_1 - n_2 + n_3)/3$ and the scaling dimensions are given by:

$$\Delta_n = \frac{1}{3}n_0^3 + n_1^2 - \frac{1}{3}n_2^2 + n_3^2 + \frac{4}{3}n_1 n_3 - \frac{8}{3}n_2(n_1 - n_2 + n_3). \tag{8}$$

It is interesting to investigate the most relevant particles content in the inner channel, we index them as $\chi_{n_1 n_2 n_3}$. There is one excitation with $e^* = 1/3$ and $\Delta = 1/3$ that is decoupled from neutral modes:

$$\chi_{111} = \frac{\phi_c}{\sqrt{3}} \tag{9}$$

also there are three "neutralons" with $e^* = 0$ and $\Delta = 2/3$:

$$\chi_{110}, \chi_{10-1}, \chi_{011} \tag{10}$$

and conjugate. Interestingly there are six quasi-particles with $e^* = 1/3$ and $\Delta = 1$ (free fermion scaling dimension):

$$\chi_{001}, \chi_{100}, \chi_{012}, \chi_{210}, \chi_{122}, \chi_{221} \tag{11}$$

These could be a signature of $\nu = 1$ like condensate (plasmon waves on the boundary of inner condensate of $e^* = 1/3$ quasi-particles). These last quasi-particles (11) have non-zero charge and are coupled to the upstream modes. Therefore they are responsible for the dominant contribution to the thermo-electric effects upstream.

Quantitative results: Thermo-electric current

Upstream thermo-electric current through a single level with energy ϵ_0 can be estimated as

$$I_{\text{th-el}} \propto [f_D(\epsilon_0) f_U(-\epsilon_0) - f_U(\epsilon_0) f_D(-\epsilon_0)] \tag{12}$$

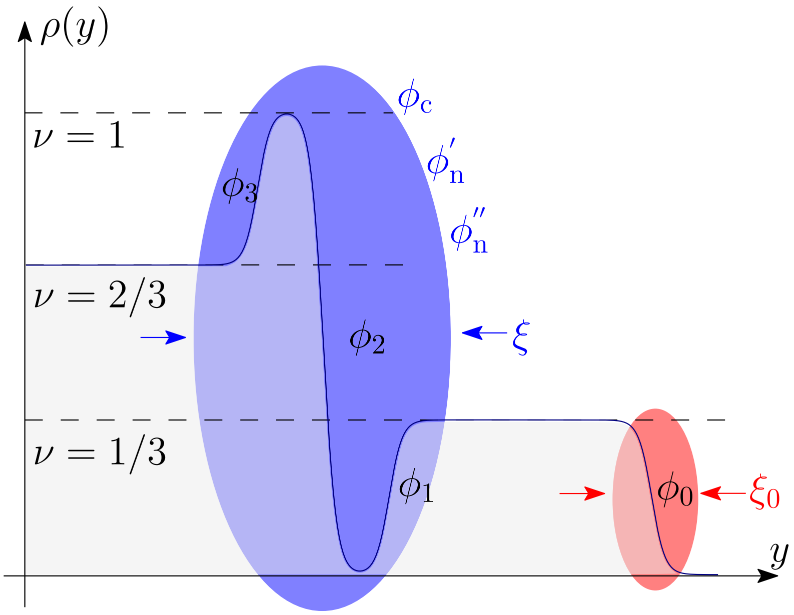

Supplementary Figure 4. Theoretical edge state structure: Schematic of the effective edge model. The blue curve shows possible transversal profile of charge density near the boundary of two-dimensional electron gas. In the effective theory, the inner modes are described in a universal way by three boson fields $\phi_\mathrm{c}, \phi_\mathrm{n}', \phi_\mathrm{n}''$ with action (4). Note that these modes have larger width than the outermost charged mode.

Here effective occupation numbers $f_i(\epsilon_0)$ are simply Fourier transforms of the corresponding correlation functions:

$$f(\epsilon_0) = \int dt e^{i\epsilon_0 t} K(t) \tag{13a}$$

$$K(t) = \langle \exp[-i\chi_\mathrm{n}(t)] \exp[i\chi_\mathrm{n}(0)] \rangle \tag{13b}$$

We take into account that downstream modes on the upper edge of quantum dot originate from a cold reservoir with base temperature T_0, while upstream modes are from "hot" Ohmic contact with temperature T_1. Assuming that the dominant cooling mechanism is by four edge states [9, 10], and taking into account that every chiral bosonic mode at temperature T carries a heat flux $\pi T^2/12$ we could write down the heat balance equation between the heat produced in the contact and heat dissipated (carried away by edge modes):

$$I\Delta\mu = \frac{\pi}{3}\left(T_1^2 - T_0^2\right). \tag{14}$$

Therefore we find that $T_1 = \sqrt{T_0^2 + (\Delta\mu/\pi)^2}$ for $\nu = 2/3$ and we have:

$$f_U(\epsilon_0) = \int dt e^{i\epsilon_0 t} \left[\frac{T_0}{\sinh(\pi T_0(t - i\eta))}\right]^{\delta_0} \left[\frac{T_1}{\sinh(\pi T_1(t - i\eta))}\right]^{\delta_1}. \tag{15}$$

In our model $\delta_0 = 1/3, \delta_1 = 2/3$ and $\Delta = \delta_0 + \delta_1 = 1$. For the lower edge effective occupation coincides with free fermions, since $\Delta = 1$:

$$f_D(\epsilon_0) = \int dt e^{i\epsilon_0 t} \frac{T_0}{\sinh(\pi T_0(t - i\eta))} = \frac{1}{e^{\epsilon_0/T} + 1} \tag{16}$$

We could also estimate the upper effective occupation (13) taking into account that at small energies the integral comes from the large times, where the correlation function decays exponentially:

$$K(t) \simeq \exp[-\pi T_\mathrm{eff}|t|], \quad T_\mathrm{eff} = \frac{T_0 + 2T_1}{3}. \tag{17}$$

Thus one could make an estimation:

$$f_U(\epsilon_0) \sim \frac{1}{e^{\epsilon_0/T_{\text{eff}}} + 1} \tag{18}$$

Results of this approximation as compared to exact numeric integration are shown in Fig 2d in the main text . They also agree well with the experimental data.

The asymptotic behavior of the thermo-electric current (12) in effective temperature approximation is

$$I_{\text{th-el}} \sim \Delta\mu^2/T_0^2, \quad \Delta\mu \ll T_0 \tag{19a}$$

$$I_{\text{th-el}} \sim \text{const}, \quad \Delta\mu \gg T_0 \tag{19b}$$

The saturation constant itself does not depend on base temperature T_0 when it is sufficiently low, but is suppressed as $\delta\epsilon_0/T_0$ when temperature becomes comparable with the level spacing $\delta\epsilon_0$ of the quantum dot.

Qualitative discussion: QPC charge and neutral transmissions

The key ingredient for the results of previous section is the electron-like scaling behavior of fractional quasi-particles (11). Here we also use the argument that the fractional quasi-particles coupled to neutral modes behave like free electrons. It is important to note that the effective action (4) is an intermediate fixed point, it has relevant neutralons $\Delta = 2/3$. In contrast, low-energy fixed point of Polchinski-Kane-Fisher model [11] has only irrelevant neutralons with $\Delta = 2$. However, quantum point contacts and quantum dots can give additional energy scale that pins the intermediate fixed point.

There are three wide inner states in our model: one charged and two upstream neutral and one narrow charged outer. The transmission of a single channel could be modeled in the quasi-classical approximation [12, 13] as

$$t_i(V) = \frac{1}{1 + e^{(V_i - V)/\delta V_i}} \tag{20}$$

where $i = \text{in}, \text{out}$. In our model there are two channels that are spatially separated, therefore they will be pinched off at different QPC voltages:

$$t_{\text{charge}}(V) = \frac{1}{2}\left[\frac{1}{1 + e^{(V_{\text{in}} - V)/\delta V_{\text{in}}}} + \frac{1}{1 + e^{(V_{\text{out}} - V)/\delta V_{\text{out}}}}\right] \tag{21}$$

Analogously, both neutral modes are located at inner channel, so that:

$$t_{\text{neutral}}(V) = \frac{1}{1 + e^{(V_{\text{in}} - V)/\delta V_{\text{in}}}} \tag{22}$$

It is natural to assume that $\delta V_{\text{in}}/\delta V_{\text{out}} \propto \xi/\xi_0 \gg 1$.

- Appearance of quasi-particles with charge $e^* = 1/3$ but with electronic scaling dimension $\Delta = 1$.
- Couplings of the above quasi-particles to the changed and neutral modes are universal $\delta_0 = 1/3$ and $\delta_1 = 2/3$ in the limit of strong Coulomb interactions.
- Inner and outer edge channels have significantly different widths $\xi/\xi_0 \gg 1$.
- Upstream neutral excitation only appear in the inner channel.

Most of these features will appear in all other filling fractions that allow hierarchic condensates. And indeed, the experimental data for $\nu = 3/5$ show that all the results are very similar.

Supplementary References

[1] Vivek Venkatachalam, Sean Hart, Loren Pfeiffer, Ken West, and Amir Yacoby. Local thermometry of neutral modes on the quantum Hall edge. *Nature Physics*, 8(9):676–681, aug 2012.

[2] Itamar Gurman, Ron Sabo, Moty Heiblum, Vladimir Umansky, and Diana Mahalu. Dephasing of an electronic two-path interferometer. *Physical Review B*, 93(12):121412, mar 2016.

[3] R. B. Laughlin. Anomalous Quantum Hall Effect: An Incompressible Quantum Fluid with Fractionally Charged Excitations. *Physical Review Letters*, 50(18):1395–1398, may 1983.

[4] S M Girvin R.E. Prange. See Chapter 7 in "The Quantum Hall Effect". *Springer, New York*, 93, 1987.

[5] B I Halperin. Quantized Hall conductance, current-carrying edge states, and the existence of extended states in a two-dimensional disordered potential. *Physical Review B*, 25(4):2185–2190, 1982.

[6] X. Wen. Chiral Luttinger liquid and the edge excitations in the fractional quantum Hall states. *Physical Review B*, 41(18):12838–12844, jun 1990.

[7] J Frohlich and A Zee. Large scale physics of the quantum hall fluid. *Nuclear Physics B*, 364(3):517–540, 1991.

[8] Ivan P Levkivskyi. Universal nonequilibrium states at the fractional quantum Hall edge. *Physical Review B*, 93(16), 2016.

[9] So Takei and Bernd Rosenow. Neutral mode heat transport and fractional quantum Hall shot noise. *Physical Review B*, 84(23), 2011.

[10] Artur O Slobodeniuk, Ivan P Levkivskyi, and Eugene V Sukhorukov. Equilibration of quantum Hall edge states by an Ohmic contact. *Physical Review B*, 88(16):165307, oct 2013.

[11] C. L. Kane, Matthew P. A. Fisher, and J. Polchinski. Randomness at the edge: Theory of quantum Hall transport at filling ν=2/3. *Physical Review Letters*, 72(26):4129–4132, jun 1994.

[12] L D Landau and E M Lifshitz. Course of Theoretical Physics - Vol 3 Quantum Mechanics - nonrelativistic theory 3rd ed, 1977.

[13] M Buttiker. Quantized transmission of a saddle-point constriction. *Physical Review B*, 41(11):7906–7909, 1990.

[14] Wang, J., Meir, Y. & Gefen, Y. Edge reconstruction in the ν=2/3 fractional quantum Hall state. *Physical review letters* 111, 246803 (2013).
